# Population genetics and sympatric divergence of the freshwater gudgeon, *Gobiobotia filifer*, in the Yangtze River inferred from mitochondrial DNA

**DOI:** 10.1002/ece3.5746

**Published:** 2019-12-15

**Authors:** Dengqiang Wang, Lei Gao, Huiwu Tian, Weiwei Dong, Xinbin Duan, Shaoping Liu, Daqing Chen

**Affiliations:** ^1^ Yangtze River Fisheries Research Institute Chinese Academy of Fishery Science Wuhan China; ^2^ School of Life Science Southwest University Chongqing China

**Keywords:** Cyt *b*, genetic structure, *Gobiobotia filifer*, sympatric population

## Abstract

The ecosystem and Pleistocene glaciations play important roles in population demography. The freshwater gudgeon, *Gobiobotia filifer*, is an endemic benthic fish in the Yangtze River and is a good model for ecological and evolutionary studies. This study aimed to decode the population structure of *G. filifer* in the Yangtze River and reveal whether divergence occurred before or after population radiation. A total of 292 specimens from eight locations in the upper and middle reaches of the Yangtze River were collected from 2014 to 2016 and analyzed via mitochondrial DNA Cyt *b* gene sequencing. A moderately high level of genetic diversity was found without structures among the population. However, phylogenetic and network topology showed two distinct haplotype groups, and each group contained a similar proportion of individuals from all sampled sites. This suggested the existence of two genetically divergent source populations in *G. filifer*. We deduced that a secondary contact of distinct glacial refugia was the main factor creating sympatric populations of *G. filifer*, and climate improvement promoted population expansion and colonization.

## Graphical abstract

The results revealed two sympatric populations coexisting in all geographical locations, and similar proportions of sympatric populations among the geographical populations. It appeared that ecosystem and historical event played important role to shape the population patterns.

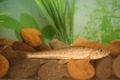

## 1 INTRODUCTION

The Yangtze River is the longest river in China and the third longest in the word, and it occupies various types of ecosystems. According to the river characteristics, the river section above Yichang City, Hubei Province, is considered the upper reaches, and the section below Yichang City the middle and lower reaches. There are approximately 361 fish species inhabiting the river basin, 177 of which are endemic (Fu, Wu, Chen, Wu, & Lei, 2003). Distribution patterns across the river are different for different fishes, and many fishes are only endemic in special sections or tributaries. Consequently, the genetic structures among populations are important for interpreting adaptation to heterogeneous environments. Additionally, the Pleistocene glaciations have had profound effects on the phylogeography and historical demography of species (Dynesius & Jansson, 2000; Hewitt, 2000). Freshwater fishes were restricted in rivers or lakes and experienced a fluctuant landscape of intermittently connected and isolated watersheds and glacial refugia, which provides many opportunities to study phylogeography and speciation (Ruskey & Taylor, 2016; Ruzzante, Walde, Macchi, Alonso, & Barriga, 2011; Taylor, 1999; Zhou, Song, Wang, Jie, & Gao, 2016).

The subfamily *Gobiobotinae* (Cyprinidae, Cypriniformes) is a group of small freshwater gudgeon distributed in the rivers of East Asia in Korea and China (Chen, 1998). Only two genera, *Gobiobotia* and *Xenophysogobio*, with 17 species belong to this subfamily, and all the fishes are benthic. He (1991) examined the skeletons and classified *Xenophysogobio* as a primitive species and *Gobiobotia filifer* as a specialized species. Wang, He, and Chen (2002) used mitochondrial DNA (mtDNA) data to construct the phylogenetic relationship that supported this idea. They also inferred that *Gobiobotinae* originated in the upper reaches of the Yangtze River. At present, four fishes, including *X. boulengeri*, *X. nudicorpa*, *G. abbreviata*, and *G. filifer*, live in the upper reaches of the Yangtze River. However, except for these four fishes, sympatric habitation among *Gobiobotinae* fishes is rare, indicating that they could have quickly adapted and specialized to new ecological environments.

**Figure 1:**
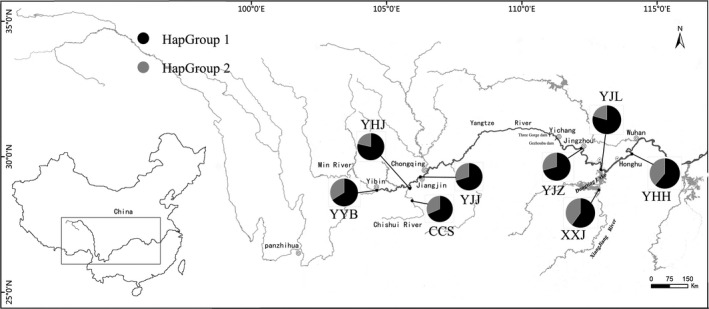
Map showing the eight sampling locations (black dots), sample sizes, abbreviated as in Table 1, with the relative frequency of the two HapGroups (HapGroup 1 and HapGroup 2)

*Gobiobotia filifer* is endemic to the Yangtze River basin (Chen, 1998), and the limits of its native range extend from the Yibin City to downstream, including tributary rivers such as Min River, Chishui River, Hanjiang River, and Xiangjiang River (Figure 1). This fish produces drifting eggs in spawning reason (Liu et al., 2018; Tian et al., 2017) and feeds on benthic organisms. The Gezhouba Dam was completed in 1988 in Yichang City, and then, the Three Gorges Dam with 175 m of water deep was completed in 2006, which form physical and ecological barriers to genetic exchange among populations. Recent surveys found that the abundance of *G. filifer* in upper Yangtze River was relatively high comparing with that before the construction of the Three Gorges Dam (Fan, Ba, & Duan, 2012; Xiong, Liu, Duan, Liu, & Chen, 2015; Yang, Xin, Ma, Kong, & Liu, 2010). However, it was less common in section and tributary rivers below the dams now (Fan et al., 2012).

Own to the specialized position in phylogeny and relatively broad distribution, *G. filifer* is a good mode for studying the local adaptation and the effect of dam on fish. For the last decades, mitochondrial DNA has been proven to be an important tool in population history, biogeography, genetic structure, and species delimitation (Hebert, Penton, Burns, Janzen, & Hallwachs, 2004; Moore, 1995). In this study, we used cytochrome *b* (Cyt *b*) to examine population genetic diversity of *G. filifer*. We aimed to (a) reveal whether genetic divergence occurred within the population and (b) determine whether divergence occurred before or after population radiation.

## 2 MATERIALS AND METHODS

### 2.1 Sampling and DNA extraction

A total of 292 individuals of *G. filifer* were collected from eight locations from 2014 to 2016; four locations were in the upper reaches of the Yangtze River and four in the middle reaches (Table 1; Figure 1). A small fin sample was clipped and preserved in 95% ethanol for each specimen.

**Table 1:** Sampled sites and sizes, population genetic parameters, and neutrality tests for the *Gobiobotia filifer* populations

Sampling location	Abbreviation	Geographical coordinates	Sample size	Haplotype number	Haplotype diversity, *H*_d_	Nucleotide diversity, *π*	Tajima's *D*	Fu's *F*s
Yibin City, Yangtze River	YYB	28°76′N 104°63′E	67	18	0.877	0.0076	0.9775	0.171
Hejiang County, Yangtze River	YHJ	28°81′N 105°83′E	14	3	0.692	0.0052	0.7419	6.439
Jiangjin City, Yangtze River	YJJ	29°27′N 106°28′E	70	17	0.815	0.0064	0.7364	−0.109
Chishui City, Chishui River	CCS	28°58′N 105°69′E	54	15	0.827	0.0068	0.5177	0.474
Jingzhou City, Yangtze River	YJZ	30°30′N 112°24′E	24	16	0.946	0.0080	−0.1841	−3.530*
Jianli County, Yangtze River	YJL	29°53′N 112°93′E	31	14	0.886	0.0068	−0.0845	−0.928
Honghu City, Yangtze River	YHH	30°07′N 114°01′E	18	10	0.935	0.0083	0.8021	0.384
Miluo City, Xiangjiang River	XXJ	28°85′N 112°89′E	14	13	0.989	0.0088	0.2064	−4.737**
	HapGroup 1		203	34	0.803	0.0020	−2.1016*	−27.182**
	HapGroup 2		89	32	0.891	0.0025	−1.4959	−27.454**
	Total		292	66	0.893	0.0072	−0.736	−28.076**

* Significant level at *p* < .05.

** Extremely significant at *p* < .01.

Genomic DNA was extracted using an easy‐DNA Kit (Omega) following the manufacturer's instructions.

### 2.2 PCR and sequencing

The cytochrome *b* (Cyt *b*) gene was amplified using polymerase chain reaction (PCR) with primers L14742 and H15915 (Xiao, Zhang, & Liu, 2001). Each 50 μl PCR contained 1–10 ng of template DNA, 5 μl of 10 × PCR buffer, 1 μl of dNTP mix (10 mM), 10 pmol of each primer, and 2 U of rTaq polymerase (TaKaRa). PCRs were conducted in a thermal cycler (T100; Bio‐Rad) with the following program: one cycle of denaturation at 95°C for 4 min; 30 cycles at 94°C for 30 s, 56°C for 30 s, and 72°C for 90 s; and one final cycle at 72°C for 10 min. Successful PCR products were separated by electrophoresis on a 1.0% agarose gel and purified using the Agarose Gel Purification Kit (Qiagen).

Several samples with distinct phylogenetic clades were also used to test the existence of cryptic species using COI gene. The primers and PCR information were as Ward, Zemlak, Innes, Last, and Hebert (2005).

Purified products then were sequenced with an ABI PRISM 3730 sequencer. Sequencing primers were the same as those used for PCR amplification. All unique sequences have been deposited in GenBank.

### 2.3 Data analyses

Sequences were assembled by Lasergene v7.1 (http://www.dnastar.com/) and aligned with the Clustal X 1.81 program (Thompson, Gibson, Plewniak, Jeanmougin, & Higgins, 1997). Population genetic diversity was measured for all samples, sampling groups, and divergent groups by determining haplotype diversity (*h*) and nucleotide diversity (*π*) in the DnaSP 6.0 software (Rozas et al., 2017).

A median‐joining haplotype network was constructed in Network v4.6 (http://www.fluxus-engineering.com/). Phylogenetic analysis of haplotypes was conducted using the neighbor‐joining (NJ) method and Bayesian inference (BI). The NJ analysis with the Kimura 2‐parameter distance method was carried out in MEGA 7.0 (Kumar, Stecher, & Tamura, 2016). The tree nodes and branch lengths were statistically tested using the bootstrap method with 1,000 replicates and an interior branch test, respectively. BI analysis was carried out using MrBayes v3.1.2 (Ronquist & Huelsenbeck, 2003). Best‐fit models for the Bayesian analysis were inferred by hierarchical likelihood ratio tests on the IQ‐TREE Web server (http://iqtree.cibiv.univie.ac.at/).

Pairwise *F*_ST_ values and analysis of molecular variance (AMOVA) were used to assess the population configuration in Arlequin v3.5 (Excoffier & Lischer, 2010). The pairwise *F*_ST_ values between sites were calculated and assessed for significance by comparison with 10,000 permutations of data.

Migration patterns were estimated using the coalescent‐based program MIGRATE‐n v3.6.11, which estimates migration rates between two groups of populations—groups with four populations in upper reaches (UPG) and four populations in middle reaches (MPG). Estimation of parameters in MIGRATE was done using Bayesian approach (Beerli, 2006).

Historical demographic expansions were investigated with neutral tests, such as Tajima's *D* (Tajima, 1989) and Fu's *F*s (Fu, 1997), and pairwise nucleotide mismatch distributions; all of these tests were implemented in DnaSP 6.0 (Rozas et al., 2017). Departures from neutrality of Fu's *F*s and Tajima's *D* test indicate recent population expansions under assumptions of neutrality (Ramos‐Onsins & Rozas, 2002). The demographic history of *G. filifer* was explored using mismatch analysis of Cyt *b* mitochondrial sequences. This method is based on the premise that population growth or decline leaves distinctive signatures in DNA sequences compared to constant population size. To compare the observed distributions with those expected under the expansion model, we calculated the sum of square deviation (SSD) and the Harpending's raggedness index (Harpending, 1994; Rogers & Harpending, 1992). To test the significance of the observed data from the simulated data with sudden expansion, 100 bootstrap replicates were applied. If the sudden expansion model was not rejected, the equation *τ* = 2*µt* was used to estimate the approximate expansion time, where *τ* is the date of the growth or decline measured in units of mutational time calculated in Arlequin v3.5 (Excoffier & Lischer, 2010), and *u* is the mutation rate per sequence and per generation. The *u* was calculated from *u* = 2*μk*, where *μ* is the substitution rate per site, and *k* is the number of nucleotides of the analyzed fragment (1,009 bp in this study).

## 3 RESULTS

A Cyt *b* dataset of 1,009 bp in size was obtained, and a total of 57 polymorphic sites were detected including 14 singleton variable and 43 parsimony informative sites. Among 292 sequences, 66 unique haplotypes were determined. The mean haplotype diversity and nucleotide diversity were 0.893 (range from 0.692 to 0.989) and 0.0072 (range from 0.0052 to 0.0088; Table 1), respectively. Genetic distance among haplotypes ranges from 0.001 to 0.019 (Table S1).

Both of the phylogenetic topologies of 66 *G. filifer* mtDNA haplotypes generated with NJ and Bayesian inference were similar, which are presented with two distinct haplotype groups, HapGroup 1 and HapGroup 2 with high confidence values (Figure S1). The sample distributions in the groups were not consistent with the geographical locations, and the two populations could each be found at all locations (Table S2).

**Figure 2:**
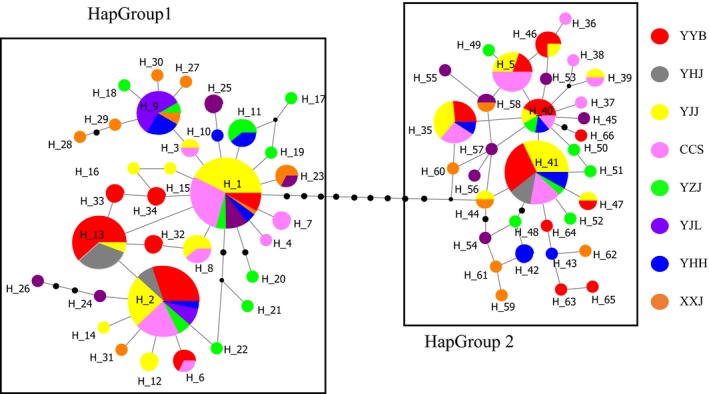
A median‐joining network of 66 Cyt *b* haplotypes of *Gobiobotia filifer* in this study. The circle size is proportional to the haplotype frequency. The black dots indicate mutation steps

Haplotype median‐joining network supported the phylogenetic tree result that all of the mtDNA haplotypes fell into two haplotype groups (Figure 2). All haplotypes from each group were separated by at least 11 mutation steps, whereas neighbor haplotypes differed by a maximum of four (HapGroup 1) and two (HapGroup 2) mutations within groups.

HapGroup 1 had 34 haplotypes and contained 203 individuals (69.5% of samples), whereas HapGroup 2 had 32 haplotypes and 89 individuals (30.5% of samples; Table S3). The proportion of sample size of HapGroup 1 to HapGroup 2 was 2.28, and there was no significant difference among the proportions in eight sampling locations with *p* = .63 for the *t* test (two‐tailed) and *p* = .93 for the chi‐square test (Table S3). Haplotypes 2 (63 individuals, 31.0%) and 1 (61, 30.0%) were the most common haplotypes in HapGroup 1, and haplotype 41 was the most common in HapGroup 2 (25 individuals, 28.1%). The former two haplotypes were shared by seven locations, and the last one was shared by six locations. No single haplotype was found in all locations. The haplotype diversity and nucleotide diversity of HapGroup 1 and HapGroup 2 were 0.803 and 0.0020 and 0.891 and 0.0025, respectively.

The global AMOVA showed that no significant differentiation occurred among geographical populations with *F*_ST_ = 4.55% (Table 2). Pairwise *F*_ST_ values further revealed that genetic differences between YHJ and other populations were moderately large and significant. The divergence between XXJ and the four populations in the upper reaches was also significant (Table 3). The migration rates described genetic migration patterns dominated by asymmetric gene flow which UPG supplies much more migrants to MPG than otherwise (M_UPG→MPG_ = 869.7; M_MPG→UPG_ = 86.8; Table 4).

**Table 2:** Analysis of molecular variance (AMOVA) among *Gobiobotia filifer* populations

Source of variation	*df*	Sum of squares	Variance component	Percentage of variation
The whole sample				
Among populations	7	7.97	0.02	4.55
Within populations	284	122.03	0.43	95.45
*F*_ST_ :0.0455				
Two groups (upper and middle reaches)				
Among groups	1	2.17	0.0098	2.15
Among populations within groups	6	5.80	0.0156	3.43
Within populations	284	122.03	0.4297	94.43
*F*_SC_: 0.0349				
*F*_ST_: 0.0557				
*F*_CT_: 0.0215				

**Table 3:** The *F*_ST_ values among *Gobiobotia filifer* populations

	YYB	YHJ	YJJ	CCS	YJZ	YJL	YHH	XXJ
1								
2	0.0183							
3	0.0522	0.1455**						
4	0.0454	0.1444**	−0.0105					
5	0.0334	0.1210**	0.0454	0.0365				
6	0.0562	0.1469**	0.0358	0.0315	0.0214			
7	0.0375	0.1273**	0.0318	0.0304	−0.0129	−0.0073		
8	0.0673*	0.1593**	0.0830*	0.0780*	0.0218	0.0269	0.0152	

* Significant level at *p* < .05.

** Extremely significant at *p* < .01.

**Table 4:** Posterior distribution of migration rate between *Gobiobotia filifer* populations in upper and middle reaches using Bayesian analysis

Parameter	2.5%	25.0%	Mode	75.0%	97.5%	Median	Mean
Θ_UPG_	0.00493	0.00700	0.00830	0.00967	0.01247	0.00857	0.00862
Θ_MPG_	0.01220	0.01793	0.02183	0.02627	0.03807	0.02337	0.02427
M_MPG→UPG_	0	0	4.3	64.0	248.7	64.3	86.8
M_UPG→MPG_	650.7	889.3	980.3	996.7	1,000.0	894.3	869.7Θ

Θ, effective population size; M, immigration rate.

The local and global neutral test showed that only HapGroup 1 had a significantly negative value for Tajima's *D*, and three populations, including XXJ, HapGroup 1, and HapGroup 2, and the total sample had highly significant negative values for Fu's *F*s (Table 1). We further tested the two HapGroups with mismatch distribution under the sudden expansion model and found similar *τ* values for the HapGroup populations. Based on *μ* = 0.76% for cyprinid fish Cyt *b* gene (Zardoya & Doadrio, 1999), the expansion times were estimated at approximately 14.3 (6.1–38.0, CI = 95%) and 16.5 (8.3–20.6, CI = 95%) 1,000 years ago for the HapGroup 1 and HapGroup 2 populations, respectively (Table 5).

**Table 5:** Mismatch analyses based on mtDNA Cyt *b* sequences for HapGroup 1 and HapGroup 2 and estimates of population expansion time

Populations	*τ* (95% intervals)	SSD (*p*‐value)	HRag (*p*‐value)	*T* (1,000 years ago)
HapGroup 1	2.195 (0.930, 5.832)	0.0062 (.35)	0.0390 (.5)	14.3 (6.1, 38.0)
HapGroup 2	2.537 (1.268, 3.164)	0.0051 (.05)	0.0416 (.45)	16.5 (8.3, 20.6)

Eighty‐five COI sequences with 570 bp in size were also obtained and identified to 11 unique haplotypes. Genetic distance among haplotypes ranges from 0.002 to 0.022 (Table S4). The NJ tree constructed from the haplotypes was shown similar topology as that of Cyt *b* (Figure S2).

## 4 DISCUSSION

### 4.1 Genetic diversity and structure

MtDNA Cyt *b* gene sequences have been used to study population genetics of some fishes in the Yangtze River, for example, *Zacco platypus* (Perdices, Cunha, & Coelho, 2004), *Leiocassis longirostris* (Xiao, Xia, & Ma, 2012; Yang, Xiao, Yu, & Xu, 2012), *Leptobotia elongata* (Tian, Duan, Wang, Liu, & Chen, 2013), *Siniperca chuatsi* (Tian et al., 2015), and *Saurogobio dabryi* (Li, Tang, Yu, & Liu, 2016). Compared to these fishes, the genetic diversity of *G. filifer* is currently high. Although the *F*_ST_ test did not detect genetic differentiation among the sampled populations, the pairwise analysis (Table 3) revealed moderate divergence between the YHJ population and the others (except YYB, with the minimum value; Wright, 1978). However, the river distance from YHJ to YYB, which is about 195 km, is longer than that to YJJ (about 105 km) and there are not flow barriers between these populations. It was irrational and could be a sampling error—for sampling, time from YHJ was a week, however one to several months from other sampling sites. The XXJ population also displayed moderate differentiation from the four populations in the upper reaches of the Yangtze River (Table 3) and had the highest haplotype diversity and nucleotide diversity. This population was isolated with the others by Dongting Lake and may have less exchange with other population, especially the upstream populations. However, considering the small sample size, whether the results hold true for YHJ and XXJ is still unresolved.

Two dams were constructed in Yichang City (Figure 1) in 1981 (Gezhouba Dam) and 2003 (Three Gorges Dam), and they hindered the gene flow of populations between the upper and middle reaches of the Yangtze River. We did not detect genetic differentiation among populations within groups from the upper and middle reaches (Table 2), suggesting that the dams had minimal effects on the population genetics. However, on a large scale, the genetic diversity of populations in the middle reaches was higher than that of populations in the upper reaches, and the numbers of haplotypes, site variants, and unique haplotypes in populations from middle reaches were greater than that from upper reaches. It may be the result of unidirectional gene flows that the fish could migrate from upstream to downstream across dam rather than vice versa. Such hypothesis was supported by migration rate estimation (Table 4). Historically, the Three Gorges river section of 193 km above Gezhouba Dam is narrow and very turbulent before the construction of the Three Gorges Dam and Gezhouba Dam. Such ecological condition is difficult for *G. filifer* to migrate from downstream to upstream, and dam construction has further hindered the upward migration. Therefore, ecological barriers and dam should promote the existence of higher genetic diversity in downstream reaches than in the upper reaches.

### 4.2 Sympatric population and evolutionary originally hypothesis

In this study, we found two distinct mtDNA haplotypes of *G. filifer* coexisting in all eight sampled sites, which reflects a so‐called sympatric population. Genetically different populations of fishes are commonly detected between geographical stocks because of isolation or distant barriers (Yang, Tang, et al., 2012). Sympatric populations have also been documented in several freshwater fishes including perch, walleye, rainbow smelt, and several salmonid species (Bergek & Björklund, 2007; Dupont, Bourret, & Bernatchez, 2007; Ferguson & Taggart, 1991; Lu & Bernatchez, 1999; Østbye, Næsje, Bernatchez, Sandlund, & Hindar, 2005; Palmé, Laikre, & Ryman, 2013; Pigeon, Dodson, & Bernatchez, 1998; Wilson et al., 2004). Several hypotheses have been proposed to interpret such results. First, divergent genetic lineages may reflect cryptic species with the absence of morphological diagnostic characteristics for identification. The neotropical skipper butterfly *Astraptes fulgerator* is the best example (Hebert et al., 2004). Other animals, such as the bumble bee (Scriven, Whitehorn, Goulson, & Tinsley, 2016), mollusk (Sun et al., 2016), frog (Stuart, Inger, & Voris, 2006), and fish (Borsa, Hsiao, Carpenter, & Chen, 2013; Feulner, Kirschbaum, Schugardt, Ketmaier, & Tiedemann, 2006; Rosser, 2015), were also revealed to contain sympatric cryptic species as detected through mtDNA sequence analysis. The COI sequence is usually used as DNA barcoding for species identification with the criterion of 2% or 3% sequence divergence (e.g., Costa et al., 2007; Hebert et al., 2004; Loh, Bond, Ashton, Roberts, & Tibbetts, 2014; Shen, Guan, Wang, & Gan, 2016; Ward et al., 2005). Using this sequence and the criterion, all sampled Cyprinidae fish in the midstream of the Yangtze River were successfully identified (Shen et al., 2016). However, divergent levels at 2% or 3% do not indicate valid species (April, Mayden, Hanner, & Bernatchez, 2011; Hubert et al., 2008). In *G. filifer*, the greatest genetic distance for K2P was 2.2% between haplotypes 8 and 10 of COI (Table S4), which were lower than those among species in the genus (Yang, He, Freyhof, Kai, & Liu, 2006). Therefore, it cannot be inferred that there is a cryptic species in this fish.

Second, sympatric intraspecific divergences in mtDNA could be shaped by long‐term isolation coupled to food niche and/or reproductive separation (Wimberger, 1994). Such genetic structuring is typically detected with phenotypic differences. Trophic and genetically separate sympatric populations have been reported in salmonid fishes such as Arctic char, brown trout, and whitefish inhabiting the postglacial lakes in the Northern Hemisphere, which are landlocked populations (e.g., Ferguson & Mason, 1981; Gowell, Quinn, & Taylor, 2012; May‐McNally, Quinn, Woods, & Taylor, 2015; Power, Power, Reist, & Bajno, 2009; Præbel et al., 2013; Siwertsson et al., 2013). The food content of *G. filifer* has been examined, and most of its food sources are benthic organisms, such as mosquito larvae, *Limnoperna fortunei*, and aquatic insects (Wu et al., 2008). No distinct differences in food niche or morphological characteristics were found in the *G. filifer* population.

Third, distinct lineages in the same site can also be mixed populations from geographical subpopulations through invasion or introduction. Correlations between admixture genetic lineages and invasion events have been observed in sculpins *Cottus* spp. (Nolte, Freyhof, Stemshorn, & Tautz, 2005), guppies *Poecilia reticulata* (Lindholm et al., 2005), and zander *Sander lucioperca* (Eschbach et al., 2014). However, that might not be the case for *G. filifer*. Lineages were usually detected in invaded habitats, and the proportions of population size in each lineage were varied with the scales and plasticity of the introduced population (Eschbach et al., 2014; Nolte et al., 2005). However, no barriers hindered fish migration among the sampled sites in their long history until construction of the Gezhouba Dam and Three Gorges Dam, and we did not identify any documentation about fish introduction. In fact, there is little commercial or ecological interest in introducing *G. filifer*.

Fourth, divergent populations could have originated from secondary contacts of two distinct glacial refugia. Geographical isolation with secondary contacts would predict a high level of divergence between the mtDNA haplotypes (Grant & Bowen, 1998). There are inferred examples of secondary contacts in sympatric fish populations including *Pagellus erythrinus* in the central Mediterranean Sea (Angiulli, Sola, Ardizzone, Fassatoui, & Rossi, 2016), *Rhinichthys cataractae* in the rivers of southeastern British Columbia, Canada (Ruskey & Taylor, 2016), and *Salmo trutta* in two tiny subarctic Swedish lakes (Andersson et al., 2017) and in the Loch Maree catchment, Scotland (Jacobs, Hughes, Robinson, Adams, & Elmer, 2018). This may be the most likely hypothesis for *G. filifer*. Global homogeneity implied that the two distinct populations came in contact postglacially at one position and then colonized novel environments synchronously. Population expansions (Table 5) occurred in the last glacial period (10,000–70,000 years ago), and temperature and rainfall began to increase at that time in east China (Song, Yu, & Zhu, 1998). Many fishes in the Yangtze River have been reported to experience population bottleneck followed by expansion, such as *Leiocassis longirostris* (Yang, Xiao, et al., 2012), *Squalidus argentatus* (Yang, Tang, et al., 2012), *Gymnocypris dobulai* (Chan, Li, Hu, Liu, & Xu, 2016), and *Parabramis pekinensis* (Chen et al., 2016). The asymmetric proportion of HapGroup 1 to HapGroup 2 populations could be interpreted as different founder sizes and panmixia after recontact of distinct populations, but nuclear maker is needed to support this hypothesis.

### 4.3 Concluding remarks

To our knowledge, this is the first report about sympatric genetically populations of fish in the Yangtze River. These fishes are not a cryptic species, but instead represent a secondary contact of distinct glacial refugia. This study highlights that historical and ecological factors play an important role in population patterns for fishes in the Yangtze River. *G. filifer* is likely not the only example of a sympatric population in the Yangtze River.

## CONFLICT OF INTEREST

None declared.

## AUTHORS CONTRIBUTIONS

Wang DQ, Gao L, Tian HW, Dong WW, and Duan XB collected samples. Wang DQ, Dong WW, and Duan XB performed the experiments and analyzed the data. Wang DQ, Liu SP, and Chen DQ prepared the data and wrote the manuscript. All authors read and approved the final manuscript.

## Data Availability

All DNA sequences can be accessed via GenBank accessions: MK050193‐MK050258 and MK834299‐MK834309.
