# Supplementary material for: Population genetics and sympatric divergence of the freshwater gudgeon, Gobiobotia filifer, in the Yangtze River inferred from mitochondrial DNA
Source: Ecol Evol. 2019 Dec 15;10(1):50–8. doi: 10.1002/ece3.5746 (PMC6972953; doi:10.1002/ece3.5746)
Supplement: Supplementary file 1 [file ECE3-10-50-s001.pdf]

Supporting Information

Population genetics and sympatric divergence of the freshwater gudgeon, *Gobiobotia filifer,* in the Yangtze River inferred from mitochondrial DNA

Wang DQ, Gao L, Tian HW, Dong WW, Duan XB, Liu SP, Chen DQ

This Supporting Information includes Figure S1 and S2, and Table S1, S2, S3 and S4.

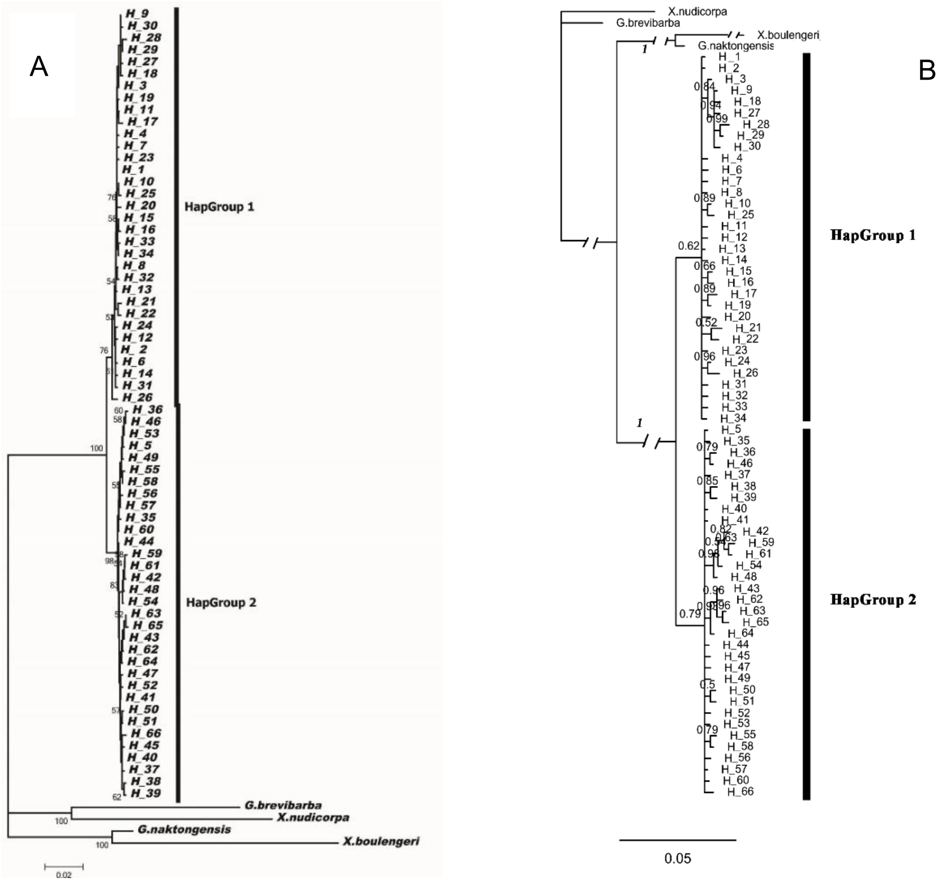

Figure S1. Phylogenetic analysis of 66 Cyt *b* haplotypes of *Gobiobotia filifer.* (A) Neighbor-joining tree with the K2P model and 1,000 bootstrap replicates. (B) Bayesian inference with the HKY+F4+G model. *G. brevibarba* (GenBank No. FJ515919), *G. naktongensis* (NC020464), *Xenophysogobio boulengeri* (KM052390) and *X. nudicorpa* (KM516103) were used as outgroups. Only bootstrap values larger than 50% are shown.

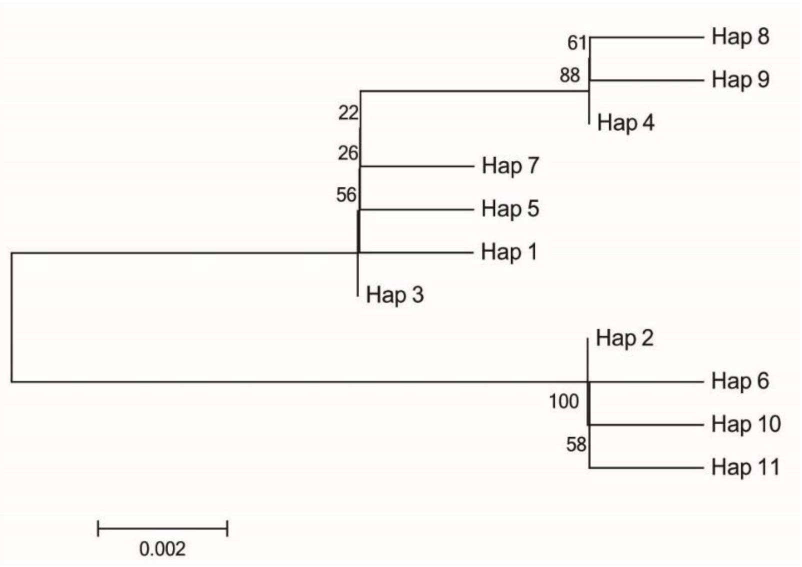

Figure S2. Neighbor-joining tree based on K2P mode with COI haplotypes of *Gobiobotia filifer*

Table S1. Pair genetic distance (%) based on K2P mode among 66 Cyt b haplotypes of *Gobiobotia filifer*.
Distances within haplotype groups (HapGroup 1 and HapGroup 2, see Table S1), with 0.1 to 0.9 percent, weren't showed. Values with 1.5 to 1.7 percent between H_5 (HapGroup 2) and haplotypes in HapGroup 1 weren't showed, either.

Haplotypes	H1	H2	H3	H4	H5	H6	H7	H8	H9	H10	H11	H12	H13	H14	H15	H16	H17	H18	H19	H20	H21	H22	H23	H24	H25	H26	H27	H28	H29	H30	H31	H32	H33	H34	H35…H66
H1																																			
⋮																																			
H34																																			
H35	1.2	1.4	1.3	1.3	0.2	1.5	1.3	1.3	1.4	1.3	1.3	1.5	1.3	1.5	1.3	1.4	1.5	1.5	1.3	1.4	1.5	1.6	1.3	1.5	1.4	1.4	1.5	1.3	1.5	1.5	1.5	1.4	1.4	1.3	
H36	1.6	1.8	1.7	1.7	0.2	1.9	1.7	1.7	1.8	1.7	1.7	1.9	1.7	1.9	1.7	1.8	1.9	1.9	1.7	1.8	1.9	2.0	1.7	1.9	1.8	1.8	1.9	1.7	1.9	1.9	1.9	1.8	1.8	1.7	
H37	1.4	1.6	1.5	1.5	0.2	1.7	1.5	1.5	1.6	1.5	1.5	1.7	1.5	1.7	1.5	1.6	1.7	1.7	1.5	1.6	1.7	1.8	1.5	1.7	1.6	1.6	1.7	1.5	1.7	1.7	1.7	1.6	1.6	1.5	
H38	1.5	1.7	1.6	1.6	0.3	1.8	1.6	1.6	1.7	1.6	1.6	1.8	1.6	1.8	1.6	1.7	1.8	1.8	1.6	1.7	1.8	1.9	1.6	1.8	1.7	1.7	1.8	1.6	1.8	1.8	1.8	1.7	1.7	1.6	
H39	1.5	1.7	1.6	1.6	0.3	1.8	1.6	1.6	1.7	1.6	1.6	1.8	1.6	1.8	1.6	1.7	1.8	1.8	1.6	1.7	1.8	1.9	1.6	1.8	1.7	1.7	1.8	1.6	1.8	1.8	1.8	1.7	1.7	1.6	
H40	1.3	1.5	1.4	1.4	0.1	1.6	1.4	1.4	1.5	1.4	1.4	1.6	1.4	1.6	1.4	1.5	1.6	1.6	1.4	1.5	1.6	1.7	1.4	1.6	1.5	1.5	1.6	1.4	1.6	1.6	1.6	1.5	1.5	1.4	
H41	1.2	1.4	1.3	1.3	0.2	1.5	1.3	1.3	1.4	1.3	1.3	1.5	1.3	1.5	1.3	1.4	1.5	1.5	1.3	1.4	1.5	1.6	1.3	1.5	1.4	1.4	1.5	1.3	1.5	1.5	1.5	1.4	1.4	1.3	
H42	1.5	1.7	1.6	1.6	0.5	1.8	1.6	1.6	1.7	1.6	1.6	1.8	1.6	1.8	1.6	1.7	1.8	1.8	1.6	1.7	1.8	1.9	1.6	1.8	1.7	1.7	1.8	1.6	1.8	1.8	1.8	1.7	1.7	1.6	
H43	1.4	1.6	1.5	1.5	0.4	1.7	1.5	1.5	1.6	1.5	1.5	1.7	1.5	1.7	1.5	1.6	1.7	1.7	1.5	1.6	1.7	1.8	1.5	1.7	1.6	1.6	1.7	1.5	1.7	1.7	1.7	1.6	1.6	1.5	
H44	1.1	1.3	1.2	1.2	0.3	1.4	1.2	1.2	1.3	1.2	1.2	1.4	1.2	1.4	1.2	1.3	1.4	1.4	1.2	1.3	1.4	1.5	1.2	1.4	1.3	1.3	1.4	1.2	1.4	1.4	1.4	1.3	1.3	1.2	
H45	1.4	1.6	1.5	1.5	0.2	1.7	1.5	1.5	1.6	1.5	1.5	1.7	1.5	1.7	1.5	1.6	1.7	1.7	1.5	1.6	1.7	1.8	1.5	1.7	1.6	1.6	1.7	1.5	1.7	1.7	1.7	1.6	1.6	1.5	
H46	1.5	1.7	1.6	1.6	0.1	1.8	1.6	1.6	1.7	1.6	1.6	1.8	1.6	1.8	1.6	1.7	1.8	1.8	1.6	1.7	1.8	1.9	1.6	1.8	1.7	1.7	1.8	1.6	1.8	1.8	1.8	1.7	1.7	1.6	
H47	1.3	1.5	1.4	1.4	0.3	1.6	1.4	1.4	1.5	1.4	1.4	1.6	1.4	1.6	1.4	1.5	1.6	1.6	1.4	1.5	1.6	1.7	1.4	1.6	1.5	1.5	1.6	1.4	1.6	1.6	1.6	1.5	1.5	1.4	
H48	1.4	1.6	1.5	1.5	0.4	1.7	1.5	1.5	1.6	1.5	1.5	1.7	1.5	1.7	1.5	1.6	1.7	1.7	1.5	1.6	1.7	1.8	1.5	1.7	1.6	1.6	1.7	1.5	1.7	1.7	1.7	1.6	1.6	1.5	
H49	1.3	1.5	1.4	1.4	0.1	1.6	1.4	1.4	1.5	1.4	1.4	1.6	1.4	1.6	1.4	1.5	1.6	1.6	1.4	1.5	1.6	1.7	1.4	1.6	1.5	1.5	1.6	1.4	1.6	1.6	1.6	1.5	1.5	1.4	
H50	1.4	1.6	1.5	1.5	0.2	1.7	1.5	1.5	1.6	1.5	1.5	1.7	1.5	1.7	1.5	1.6	1.5	1.5	1.3	1.6	1.7	1.8	1.5	1.7	1.6	1.6	1.7	1.5	1.7	1.7	1.7	1.6	1.6	1.5	
H51	1.3	1.5	1.4	1.4	0.3	1.6	1.4	1.4	1.5	1.4	1.4	1.6	1.4	1.6	1.4	1.5	1.4	1.4	1.2	1.5	1.6	1.7	1.4	1.6	1.5	1.5	1.6	1.4	1.6	1.6	1.6	1.5	1.5	1.4	
H52	1.3	1.5	1.4	1.4	0.3	1.6	1.4	1.4	1.5	1.4	1.4	1.6	1.4	1.6	1.4	1.5	1.6	1.6	1.4	1.5	1.6	1.7	1.4	1.6	1.5	1.5	1.6	1.4	1.6	1.6	1.6	1.5	1.5	1.4	
H53	1.4	1.6	1.5	1.5	0.2	1.7	1.5	1.5	1.6	1.5	1.5	1.7	1.5	1.7	1.5	1.6	1.7	1.7	1.5	1.6	1.7	1.8	1.5	1.7	1.6	1.6	1.7	1.5	1.7	1.7	1.7	1.6	1.6	1.5	

H54	1.3	1.5	1.4	1.4	0.5	1.6	1.4	1.4	1.5	1.4	1.4	1.6	1.4	1.6	1.4	1.5	1.6	1.6	1.4	1.5	1.6	1.7	1.4	1.6	1.5	1.5	1.6	1.4	1.6	1.6	1.6	1.5	1.5	1.4
H55	1.4	1.6	1.5	1.5	0.2	1.7	1.5	1.5	1.6	1.5	1.5	1.7	1.5	1.7	1.5	1.6	1.7	1.7	1.5	1.6	1.7	1.8	1.5	1.7	1.6	1.6	1.7	1.5	1.7	1.7	1.7	1.6	1.6	1.5
H56	1.3	1.5	1.4	1.4	0.3	1.6	1.4	1.4	1.5	1.4	1.4	1.6	1.4	1.6	1.4	1.5	1.6	1.6	1.4	1.5	1.6	1.7	1.4	1.6	1.5	1.5	1.6	1.4	1.6	1.5	1.6	1.5	1.5	1.4
H57	1.2	1.4	1.3	1.3	0.2	1.5	1.3	1.3	1.4	1.3	1.3	1.5	1.3	1.5	1.3	1.4	1.5	1.5	1.3	1.4	1.5	1.6	1.3	1.5	1.4	1.4	1.5	1.3	1.5	1.5	1.5	1.4	1.4	1.3
H58	1.3	1.5	1.4	1.4	0.1	1.6	1.4	1.4	1.5	1.4	1.4	1.6	1.4	1.6	1.4	1.5	1.6	1.6	1.4	1.5	1.6	1.7	1.4	1.6	1.5	1.5	1.6	1.4	1.6	1.6	1.6	1.5	1.5	1.4
H59	1.5	1.7	1.6	1.6	0.7	1.8	1.6	1.6	1.7	1.6	1.6	1.8	1.6	1.8	1.6	1.7	1.8	1.8	1.6	1.7	1.8	1.9	1.6	1.8	1.7	1.7	1.8	1.4	1.6	1.8	1.8	1.7	1.7	1.6
H60	1.1	1.3	1.2	1.2	0.3	1.4	1.2	1.2	1.3	1.2	1.2	1.4	1.2	1.4	1.2	1.3	1.4	1.4	1.2	1.3	1.4	1.5	1.2	1.4	1.3	1.3	1.4	1.2	1.4	1.4	1.4	1.3	1.3	1.2
H61	1.4	1.6	1.5	1.5	0.6	1.7	1.5	1.5	1.6	1.5	1.5	1.7	1.5	1.7	1.5	1.6	1.7	1.7	1.5	1.6	1.7	1.8	1.5	1.7	1.6	1.6	1.7	1.5	1.7	1.7	1.7	1.6	1.6	1.5
H62	1.3	1.5	1.4	1.4	0.5	1.6	1.4	1.4	1.5	1.4	1.4	1.6	1.4	1.6	1.4	1.5	1.6	1.6	1.4	1.5	1.6	1.7	1.4	1.6	1.5	1.5	1.6	1.4	1.6	1.6	1.6	1.5	1.5	1.4
H63	1.5	1.7	1.6	1.6	0.5	1.8	1.6	1.6	1.7	1.6	1.6	1.8	1.6	1.8	1.6	1.7	1.8	1.8	1.6	1.7	1.8	1.9	1.6	1.8	1.7	1.7	1.8	1.6	1.8	1.8	1.8	1.7	1.7	1.6
H64	1.3	1.5	1.4	1.4	0.3	1.6	1.4	1.4	1.5	1.4	1.4	1.6	1.4	1.6	1.4	1.5	1.6	1.6	1.4	1.5	1.6	1.7	1.4	1.6	1.5	1.5	1.6	1.4	1.6	1.6	1.6	1.5	1.5	1.4
H65	1.6	1.8	1.7	1.7	0.6	1.9	1.7	1.7	1.8	1.7	1.7	1.9	1.7	1.9	1.7	1.8	1.9	1.9	1.7	1.8	1.9	2.0	1.7	1.9	1.8	1.8	1.9	1.7	1.9	1.9	1.9	1.8	1.8	1.7
H66	1.5	1.7	1.6	1.6	0.3	1.8	1.6	1.6	1.7	1.6	1.6	1.8	1.6	1.8	1.6	1.7	1.8	1.8	1.6	1.7	1.8	1.9	1.6	1.8	1.7	1.7	1.8	1.6	1.8	1.8	1.8	1.7	1.7	1.6

Table S2. Cyt *b* Haplotype distribution across *Gobiobotia filifer* populations. Haplotypes were assigned to two groups, HapGroup 1 and HapGroup 2, based on results of Figure S1

Groups	Hlotyes	YYB	YHJ	YJJ	CCS	YJZ	YJL	YHH	XXJ	Total
	H_1	5		25	17	3	6	3	1	61
	H_2	19	5	15	13	4	5	2		63
	H_ 3			1	1					2
	H_4				1					1
	H_6	2			1					3
	H_7				2					2
	H_8			3	2					5
	H_9					1	7	3	1	12
	H_10							1		1
	H_11					3		2		5
HapGroup 1	H_12			2						2
	H_13	11	6	1						18
	H_14			1						1
	H_15			1						1
	H_16			1						1
	H_17					1				1
	H_18					1				1
	H_19					1				1
	H_20					1				1
	H_21					1				1
	H_22					1				1

	H_23						1		2	3
	H_24						1			1
	H_25						2			2
	H_26						1			1
	H_27								1	1
	H_28								1	1
	H_29								1	1
	H_30								1	1
	H_31								1	1
	H_32	2								2
	H_33	2								2
	H_34	2								2
	H_5	2		3	5					10
	H_35	3		4	3			1		11
	H_36				1					1
	H_37				1					1
	H_38				1					1
	H_39			1	1					1
HapGroup 2	H_40	3		1	1	1		1		7
	H_41	7	3	8	4	1		2		25
	H_42							2		2
	H_43							1		1
	H_44			1					1	2
	H_45			1						1
	H_46	3		1						4

H_47		1				2
H_48			1			1
H_49			1			1
H_50			1			1
H_51			1			1
H_52			1			1
H_53				1		1
H_54				1		1
H_55				1		1
H_56				1		1
H_57				1		1
H_58				1	1	2
H_59					1	1
H_60					1	1
H_61					1	1
H_62					1	1
H_63	1					1
H_64	1					1
H_65	1					1
H_66	1					1

Table S3. Numbers of HapGroup 1 and HapGroup 2 and their proportions in *Gobiobotia filifer* populations. The difference in proportions among the populations was tested by the T-test（two tailed, P=0.63）and chi-square test (P=0.93) under the null hypothesis.

	YYB	YHJ	YJJ	CCS	YJZ	YJL	YHH	XXJ	total
HapGroup 1	43	11	50	37	17	23	11	9	203
HapGroup 2	22	3	21	17	7	6	7	6	89
Rate of 1 to 2	1.96	3.67	2.38	2.178	2.43	3.83	1.57	1.5	2.28

Table S4 Pair genetic distance (%) based on K2P mode among 11 COI haplotypes of *Gobiobotia filifer*

	Hap_1	Hap_2	Hap_3	Hap_4	Hap_5	Hap_6	Hap_7	Hap_8	Hap_9	Hap_10	Hap_11
Hap_1											
Hap_2	1.6										
Hap_3	0.2	1.4									
Hap_4	0.5	1.8	0.4								
Hap_5	0.4	1.6	0.2	0.5							
Hap_6	1.8	0.2	1.6	2.0	1.8						
Hap_7	0.4	1.6	0.2	0.5	0.4	1.8					
Hap_8	0.7	2.0	0.5	0.2	0.7	2.2	0.7				
Hap_9	0.7	2.0	0.5	0.2	0.7	2.2	0.7	0.4			
Hap_10	1.8	0.2	1.6	2.0	1.8	0.4	1.8	2.2	2.2		
Hap_11	1.8	0.2	1.6	2.0	1.8	0.4	1.8	2.2	2.2	0.4	
